# WeldLight: A Lightweight Weld Classification and Feature Point Extraction Model for Weld Seam Tracking

**DOI:** 10.3390/s25185761

**Published:** 2025-09-16

**Authors:** Ang Gao, Anning Li, Fukang Su, Xinqi Yang, Wenping Liu, Fuxin Du, Chao Chen

**Affiliations:** 1 School of Mechanical Engineering, Shandong University, Jinan 250061, China; 202414363@mail.sdu.edu.cn (A.G.); 202214806@mail.sdu.edu.cn (A.L.); 202334436@mail.sdu.edu.cn (F.S.); 202314368@mail.sdu.edu.cn (X.Y.); dufuxin@sdu.edu.cn (F.D.); chaochen@sdjtu.edu.cn (C.C.); 2 Key Laboratory of High-Efficiency and Clean Mechanical Manufacture, Ministry of Education, Shandong University, Jinan 250061, China; 3 State Key Laboratory of Advanced Equipment and Technology for Metal Forming, Beijing 100083, China; 4 School of Rail Transportation, Shandong Jiaotong University, Jinan 250357, China

**Keywords:** feature point extraction, laser vision sensor, lightweight network, seam tracking, welding seam classification

## Abstract

To address the issues of intense image noise interference and computational intensity faced by traditional vision-based weld tracking systems, we propose WeldLight, a lightweight and noise-resistant convolutional neural network for precise classification and positioning of welding seam feature points using single-line structured light vision. Our approach includes (1) an online data augmentation method to enhance training samples and improve noise adaptability; (2) a one-stage lightweight network for simultaneous positioning and classification; and (3) an attention module to filter features corrupted by intense noise, thereby improving stability. Experiments show that WeldLight achieves an F1-score of 0.9668 for seam classification on an adjusted test set, with mean absolute positioning errors of 1.639 pixels and 1.736 pixels on low-noise and high-noise test sets, respectively. With an inference time of 29.32 ms on a CPU platform, it meets real-time seam tracking requirements.

## 1. Introduction

With the wide application of robots in industrial manufacturing, a welding method based on teaching and reproduction has emerged. Traditional robot welding relies on workers to manually define an a priori torch position trajectory, which is inefficient and easily affected by some time-varying dimensional errors of workpieces, such as thermal deformation [1,2]. To track the welding seam in real time and realize the automatic adjustment of the welding robots, the researchers designed some advanced sensors to obtain the spatial position information and category information of the welding seam. With the rapid development of machine vision, structured light vision has found extensive application in intelligent robots [3,4] due to its high precision advantages [5] and the rich characteristic information it provides regarding welding processes [6].

**Figure 1:**
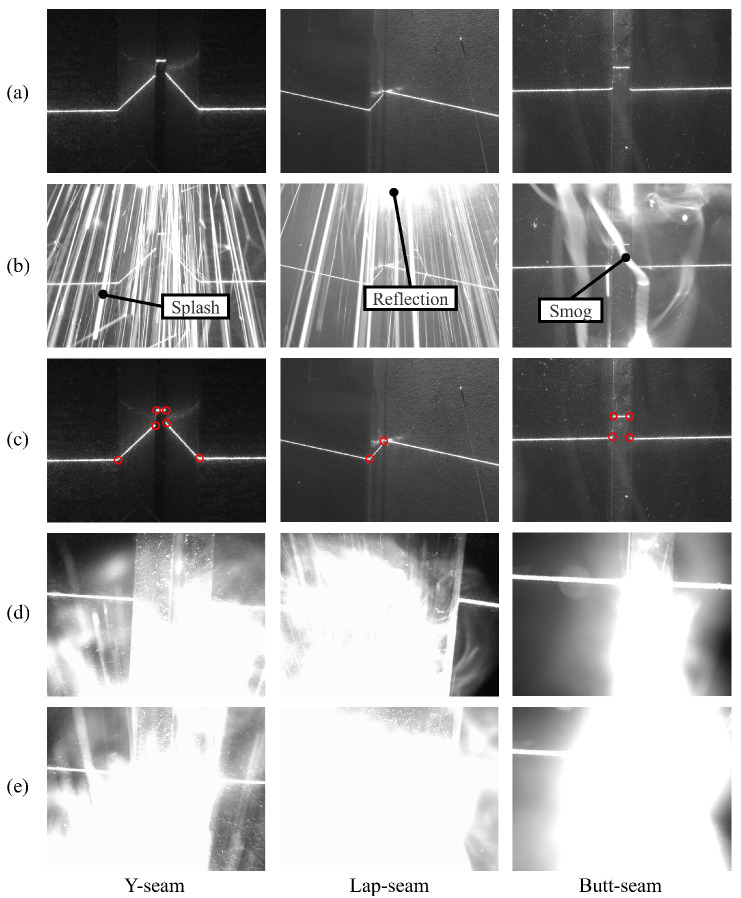
Discerning signal and noise characteristics in industrial camera images for y-seam, lap-seam, and butt-seam welds. (**a**) Noise-free structured light stripe image. (**b**) Structured light stripe image affected by noise. (**c**) Characteristic point of the structured light stripe. (**d**) Part of the feature points affected by noise. (**e**) All feature points affected by noise.

As shown in Figure 1a,c, the characteristics of single-line structured light projection stripes vary significantly based on different welding seams. This variability is valuable for acquiring position information related to welding seam feature points and facilitates the convenient classification of stripes. Such classification is crucial for adjusting the welding process parameters effectively. However, as shown in Figure 1b, common noises in the welding process, including splash, smog, and reflection light of arc, will be confused with stripes collected by industrial cameras. Several morphology-based methods have been proposed to realize the extraction of welding seam features. Li et al. [7] used a Kalman filter to track the meaningful laser stripes on the image through a window to reduce the influence of image noise during welding, and the laser stripes are decomposed into line–junction–line combination fragments to obtain the weld type and the position of feature points on the image. Yang et al. [8] applied a kernelized correlation filters’ algorithm to realize seam tracking with feature point marks, which can adapt to different types of weld seams. Two common challenges exist in morphology-based research. One is that the tasks of feature point (or region) extraction and weld classification are usually two-stage. On the flip side, while these models possess a degree of resistance to noise, their tracking accuracy degrades under sustained high-noise conditions [9].

The evolution of deep learning technology in computer vision has led to the refinement of object detection and semantic segmentation, enabled by advances in computing hardware and the development of convolutional neural networks (CNNs). These tasks are frequently transferred to seam feature extraction tasks utilizing line structured light, contributing to the development of an anti-noise model for effective feature extraction, as evidenced by existing research.

Gao et al. [10] built the YOLO-WELD model based on YOLOv5 in the task of detecting weld feature points. RepVGG is used as the backbone network, and the NAM attention mechanism and lightweight head layer, RD-Head, are introduced to improve the detection effect. Deng et al. [11] improved CenterNet, used DenseNet as the backbone network, and adjusted the head layer to separate the feature point position regression task from the weld classification task. This operation prevented the general feature detection network from associating different categories with feature points on a laser stripe image. Liu et al. [12] reported a method to extract the feature points of multi-layer and multi-pass welding seams by using a conditional generative adversarial network (CGAN) and improving the CNN model. Cacarion et al. [13] presented a detection model without NMS (non-maximum suppression) and DETR (detection transformer), which considers CNN as a feature extractor, and used an improved Transformer [14] to realize the further mining of the global information of the input picture. Because of the self-attention mechanism of the Transformer, the DETR incurs considerable computational complexity (in this paper, DETR is used as a typical algorithm to compare the inference time of the model).

However, these methods based on neural networks require considerable computing resources; in turn, they necessitate higher computing performance from the central control equipment integrated into welding robot systems. Notably, such systems commonly lack GPU acceleration support. To address this challenge, lightweight neural network techniques have been extensively explored in computer vision. Typical approaches include pruning, knowledge distillation, weight quantization, and especially structural simplification. A variety of effective lightweight backbones have emerged, ranging from MobileNet [15,16,17,18] and ShuffleNet [19,20] to newer designs such as Shvit [21] and LSNet [22]. Alongside these developments, many studies have begun integrating attention modules [23,24,25] into lightweight architectures to counterbalance the performance degradation often induced by aggressive model simplification.

Liu et al. [26] extended the concept of depthwise separable convolution from MobileNet to 3-D convolution. They combined the 2-D input image with temporal information from image sequences to assess the current welding state. Additionally, three attention mechanisms were introduced to enhance the model’s robustness against welding noise. Ma et al. [27] developed the WeldNet model specifically for welding tasks, encompassing starting point detection and seam classification. The model’s architecture employs ShuffleNet as the backbone network. However, although these methods adopt lightweight network architectures and demonstrate real-time performance on GPUs, their CPU performance remains limited, and they require large-scale training datasets, thereby hindering the cost-effective deployment of neural networks in industrial applications.

There are also some feature extraction methods based on the semantic segmentation model. Zou et al. [28] developed a lightweight laser stripe image segmentation model by replacing the backbone with ShuffleNetv2. They achieved laser stripe segmentation and improved the model by a pruning operation based on trainable parameters of the Batch Normalization (BN) layer, combined with a welding seam tracking algorithm. Despite maintaining a high inference speed on the CPU, this method does not directly provide the specific position of the welding seam in a one-stage manner.

Additionally, an efficient ViT (Vision Transformer) [29] based on cascaded grouping attention was proposed, a substitute for multi-head self-attention (MHSA). The structure of this attention mechanism can have fewer parameters and obtain higher inference speed because it is memory-efficient.

Utilizing a lightweight model in welding tasks can decrease the number of network parameters and floating-point operations (FLOPs), making it more suitable for deployment on edge computing devices with limited computational capabilities. Nevertheless, this comes at the cost of diminished performance in abstract feature extraction, potentially weakening the model’s noise robustness.

To address the issues above, the objectives of this paper are as follows:

1. To propose a one-stage model for (1) extracting the position of light stripe feature points of weld structures and (2) performing weld classification (e.g., identifying groove types) by analyzing distinctive geometric patterns in the light stripe.
2. The proposed method can be used to enhance the model’s robustness to image noise during welding without affecting the real-time performance of the model.
3. To reduce the model’s parameters and computational cost can be deployed on the computing platform where GPU acceleration is unavailable, such as an embedded industrial computer (EIC), and at the same time maintain a high inference speed to meet the real-time requirements of welding tracking systems in the industrial field.
4. Compared with typical one-stage target detection models, the study aims to test and validate the feature point positioning performance under different noise conditions, weld classification performance, and real-time performance of the proposed model.

The paper comprises five sections. Section 2 details the hardware devices and datasets employed in the study, while Section 3 provides a structural description of WeldLight. Following this, Section 4 outlines the specific configuration of the experiment and results obtained from the comparative verification of various models. Section 5 presents the obtained conclusions.

## 2. Hardware Platform and Dataset

### 2.1. Hardware Platform and Measuring Principle

**Figure 2:**
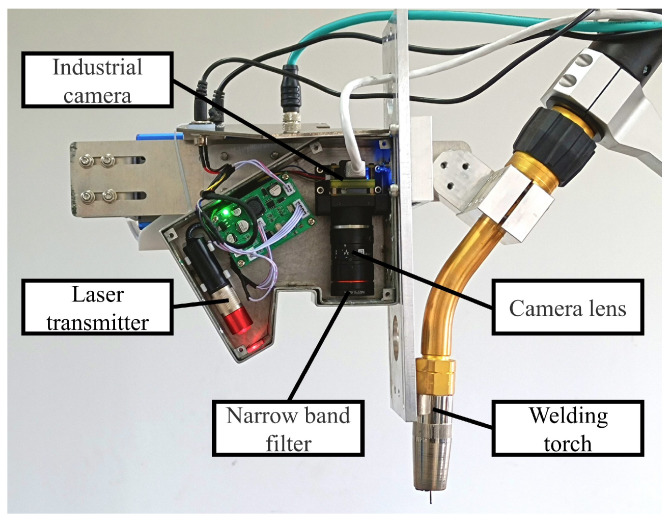
Laser vision sensor experimental platform.

As shown in Figure 2, the self-designed welding seam positioning and tracking sensor based on single-line structured light includes one industrial camera, a single-line laser transmitter, a narrow band filter, and others. The model of the used industrial camera is CB016 from HIKROBOT with a sensor size of 1/2.9^″^ and 1440 × 1080 resolution, equipped with a lens with a focal length of 25 mm. The wavelength of both the single-line laser transmitter and narrow band filter is about 660 nm.

**Figure 3:**
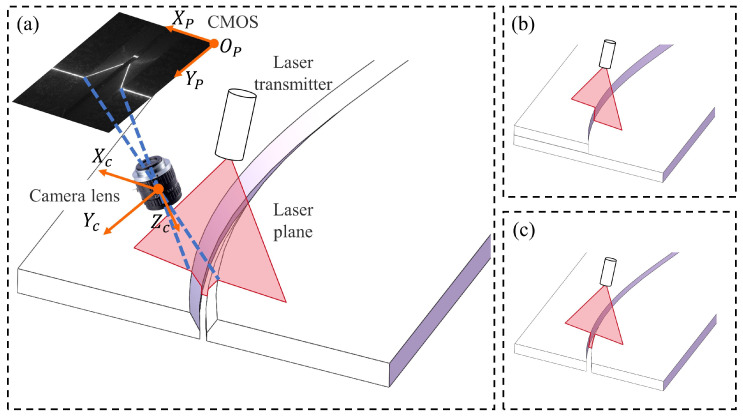
The effect diagram of structured light projection on different welds. (**a**) illustrates the projection effect on a Y-seam and embodies the measurement principle of a single-line structured light vision sensor, and (**b**,**c**) depicts the projection effect of structured light on the lap-seam and butt-seam, respectively.

As shown in Figure 3, a pixel coordinate frame (PCF) and a camera coordinate frame (CCF) are established in the line structured light imaging system illustrated in the figure; a random feature point in 2-D PCF can be transformed into 3-D CCF by

(1) $$p_{c}=z_{c}M^{-1}q_{p},$$

where $p_{c}$ is the coordinate of points on the laser stripe relative to CCF. $q_{p}$ is the 2-D coordinate of the feature point of the welding seam on the PCF, with the upper left corner of the image as the origin of the coordinate and the right and downward directions as positive, respectively. $z_{c}$ denotes the projection value on the *Z* axis of the feature point with respect to CCF, which is an unknown variable. *M* denotes the intrinsic matrix of the camera, which can be calculated by [30].

The laser plane equation of the line structured light with respect to the CCF can be determined by

(2) $$n^{T}\cdot p_{c}+d=0m$$

where {n,d} stands for the normal vector of the plane and the distance between the origin of CCF and the laser plane, respectively, which can be calculated by the calibration method proposed in [31] to obtain the general equation of the structured light plane relative to the CCF. According to (1) and (2), the coordinates of feature points concerning CCF can be represented by

(3) $$p_{c}=-\frac{d}{n^{T}M^{-1}q_{p}}M^{-1}q_{p}.$$

Equation (3) will be employed in Section 4.3 to assess the comprehensive performance of the model and hardware platform on feature point positioning.

### 2.2. Welding Image Dataset

**Figure 4:**
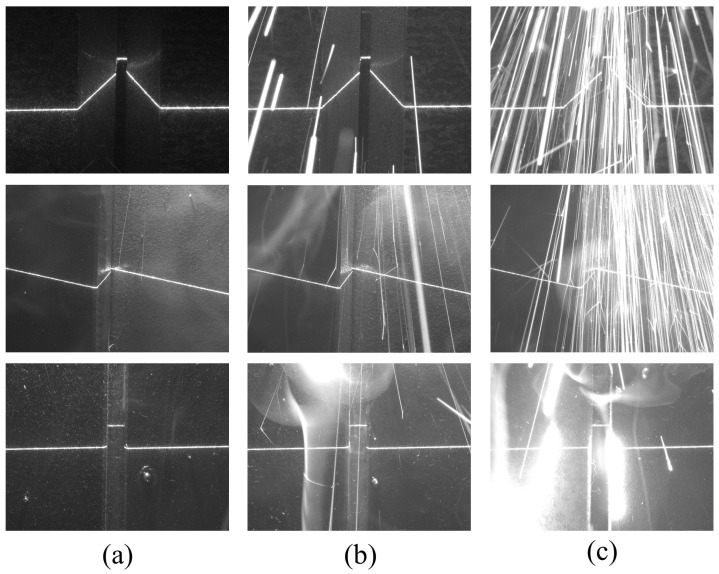
Images with different noise levels in the dataset. (**a**) Noise-free image; (**b**) slightly noisy image; (**c**) high-noise image.

In complex and variable industrial settings, neural networks often require new training data to adapt to evolving environments, and precise annotation typically demands substantial human effort. Consequently, developing models suitable for small-batch datasets becomes necessary. A total dataset of 1500 images (1440×1080 pixels), comprising Y-seam, lap-seam, and butt-seam welding stripes captured by CMOS cameras, was collected. We divided the test images into low-noise and high-noise subsets to better evaluate the robustness of the network under different operating conditions. The low-noise subset consists of noise-free and slightly noisy images, in which light stripes remain clear, and the minor disturbances can still be reliably recognized using traditional morphological methods, as illustrated in Figure 4a,b. The high-noise subset includes images with extensive splash and fume noise that severely interfere with recognition and even obscure critical features. Such cases are generally difficult to identify using morphological methods, as shown in Figure 4c, and they account for approximately 50% of the test set. The dataset was manually annotated, where the positions of occluded keypoints in the high-noise subset were estimated based on the continuity of light stripe variations.

It is worth noting that we excluded extremely noisy examples in the high-noise subset (e.g., Figure 1e), which may result from occasional extreme welding noise, highly reflective components, or hardware design limitations. In such cases, most light-stripe features become invisible, making single-image recognition ineffective. Fortunately, such extreme noise conditions are rarely encountered in typical welding scenarios. When they do arise, mitigation can be achieved through specialized hardware improvements (e.g., adding optical filters or adjusting laser wavelengths) or through downstream processing. The latter may involve trackers that integrate temporal information to predict the current occluded point or, alternatively, skipping the frame and continuing with welding path fitting. In this study, Kalman filtering was adopted for such predictions, although its details are not elaborated here.

The dataset division is detailed in Table 1. Although the evaluation in this work is restricted to three weld seam types, the approach is not confined to these cases; with appropriate specification of feature points, it can be generalized to a wider variety of seam geometries.

### 2.3. Online Data Augmentation

The quality of neural network training is highly dependent on the quality and scale of the collected dataset; however, data collection and annotation are often labor-intensive and time-consuming. Therefore, in many real-world applications, data augmentation is commonly employed to simulate variations in working environments, sensor models, and sensor positions, thereby enabling effective training on limited data. For example, previous studies [32,33] designed data augmentation techniques tailored to their respective application domains, which improved the dataset quality and enhanced the performance of neural network models.

In this study, we similarly adopt data augmentation to enhance the robustness of the neural network model. Specifically, data augmentation is applied to increase the diversity of training samples and improve the model’s generalization ability through techniques such as image flipping, warping, affine transformations, and variations in hue, saturation, and value. In addition, common noise patterns observed in welding processes are also simulated to further improve the training quality of the network.

**Figure 5:**
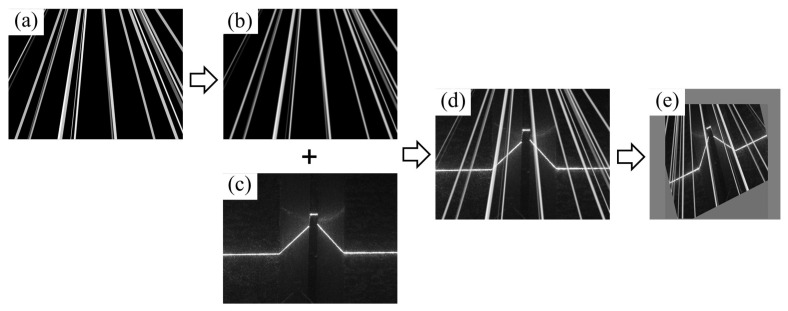
The operation flow of the proposed data augmentation method. (**a**) is a randomly generated simulated splash light noise map; (**b**) is the result of Gaussian filtering on (**a**); (**c**) is the original image from the dataset; (**d**) is the result of superimposing (**b**) on (**c**); (**e**) indicates the results of other data augmentation operations.

Splashes are the main interference factor in detecting feature points captured by the camera. To mitigate the impact of splash noise on the neural network model, a method involving the random generation of splashes is introduced into the traditional online data augmentation step to enhance the learning of splash features by the network model. So, in every epoch, the images loaded from the dataset undergo different forward passes and backward propagations. It is crucial to note that this process is distinct from the offline splash addition introduced in this work, as detailed in [10]. As illustrated in Algorithm 1, for each noise parameter, we initialize an empty splash image and generate num random lines. Each line is assigned a random starting point near the light source, a random direction, and random attributes including width and intensity. The line is then randomly segmented and rendered on the splash image. After applying Gaussian blur to the splash image, it is superimposed on the original image. Finally, the original images are blended with a noise template and subjected to a sequence of augmentation operations, including cropping, scaling, affine transformations, and brightness adjustments. The overall effect of this noise generation process is shown in Figure 5.
**Algorithm 1 **Random Noise Generation Method1:**procedure **RandomNoise(Image,num)2:    **for** each parameter *p* in noise_parameter **do**3:        splash_image←0     ▷zero matrix with same size as Image4:        **for** i←1 to num **do**5:           *P_start_*←GeneratePoint(light source region)6:           *d*←RandomDirection()7:           (*w, α*)←RandomWidthIntensity()8:           *L*←ConstrustLine(*P_start_, d, w, α*)9:           *L*←TruncateLine(*L*)10:         DrawLine(splash_image,L)11:       **end for**12:       *splash_image*←GaussianBlur*(splash_image)*13:       Image←Image+splash_image14:   **end for**15:   **return** Image16:**end procedure**

## 3. WeldLight Structure

### 3.1. Overall Structure

**Figure 6:**
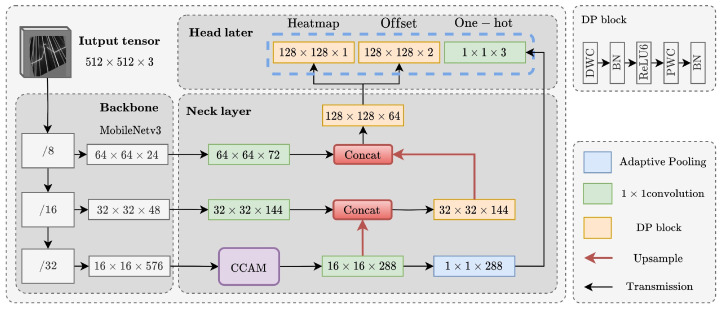
Overall layout of the proposed network.

As shown in Figure 6, WeldLight can be divided into three parts: the backbone layer, the neck layer, and the head layer.

The backbone layer is responsible for extracting features and outputting feature map branches with different downsample factors, which are then delivered to the neck layer. MobileNetV3-Small is chosen in the proposed work. In the MobileNetV3 block, a sequence consisting of a 3×3 depthwise convolution (DWC) with a BN layer and different nonlinearities (i.e., h-swish and ReLU), followed by a channel-fusing 1×1 pointwise convolution (PWC) with a BN layer, is proposed to replace one standard 3 × 3 convolution with BN and ReLU. Notably, a similar DP block (Figure 6) is used in the neck and head layers of WeldLight, employing the ReLU6 function as the nonlinearity to better suit feature-point localization. In terms of convolution computation, the quantitative relation between DP block and one standard 3 × 3 convolution is given by (4).

(4) $$\frac{k\times k\times C_{in}\times H\times W+C_{in}\times C_{out}\times H\times W}{k\times k\times C_{in}\times C_{out}\times H\times W}=\frac{1}{C_{out}}+\frac{1}{k^{2}},$$

where the numerator and denominator of the fraction represent the computations of the DP block and the standard 3×3 convolution, respectively, *k* denotes the kernel size, and $C_{in},C_{out},H,W$ denote the input channels, output channels, and the spatial dimensions of the input feature map. Generally, there are many output channels of convolution operation, especially the number of channels in the deep layer, which is much larger than the size of the convolution kernel. Consequently, the calculation can be reduced to about $\frac{1}{k^{2}}$.

In the neck layer, a feature pyramid network (FPN) [34] construction is applied. Due to many stages of backbone producing feature maps of the different resolutions with different semantic levels, the deepest three branches produced by the backbone with downsampling strides specifically of {8,16,32} with respect to the input image will be fused by FPN, as shown in Figure 6. Though more branches merged in the neck layer indicates the model can integrate deep abstract semantic information and superficial coarse-grained feature information to achieve better small-scale feature detection capability, the number of branches should not increase without limit, because more branches will seriously slow down the inference speed [20]. Through experimentation, the optimal branch number of three was determined to minimize computational complexity and latency while preserving a certain level of positioning accuracy.

**Figure 7:**
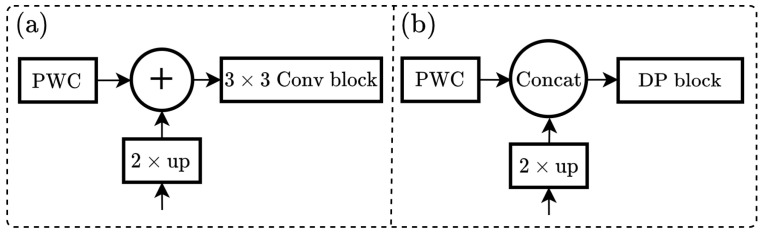
Architecture of top-down pathway and lateral connection. (**a**) is the original version of FPN; (**b**) is the proposed version.

As depicted in Figure 7b, the improved top-down pathway and lateral connection employ concatenation instead of addition, as seen in the original approach shown in Figure 7a. Channel adjustment facilitates merging the feature map from the PWC with the upsampled output of the coarser-resolution feature map containing stronger semantic information. A DP block is also utilized to substitute the standard 3×3 convolution. Ultimately, the neck layer will generate a feature map of size 128×128×64, which will subsequently be input into the head layer.

Additionally, within the neck layer, the network links a proposed attention module to the output of 32× downsampled feature map, aiming to enhance the model’s robustness against noise, as detailed in Section 3.2.

In the head layer, it is necessary to predict the position of feature points, which is realized by a heatmap tensor and an offset tensor. The task also requires classifying the laser stripes to determine the weld type, which requires the head layer to output a one-hot tensor. The standard 3×3 convolution block for decoupling the input tensor of the head layer is also replaced by a DP block. At this stage, the PWC not only fuses features across channels but also adjusts the number of channels. Since WeldLight does not classify each feature point separately but instead predicts a category for the entire laser stripe image, the categorical output of the head layer mainly derives from the lower-resolution feature map in the deepest backbone layer, enabling large-scale perception and producing a global category prediction rather than point-specific classification.

### 3.2. Cascaded Channel Attention Module (CCAM)

The deepest output shape from the backbone network obtained by FPN is $H_{in}\times W_{in}\times C_{in}$, specifically 16×16×512. It should be noted that a significant portion of the features are related to noise. These features will be merged in the neck layer and finally affect outputs from the head layer. In order to weaken the influence of noise, CCAM is proposed to filter the noise.

**Figure 8:**
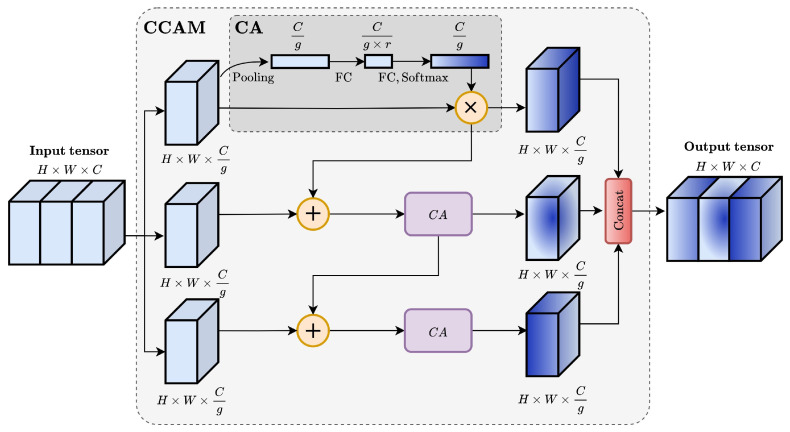
Specific structure of cascade channel attention module.

As shown in Figure 8, the core of CCAM is a channel attention (CA) block, which serves as a feature selector. Initially, a global average pooling layer is used to reduce the input feature map to a single vector, which is then fed to the first fully connected (FC) layer to reduce its dimensionality. A second FC layer restores the feature dimension. The resulting vector is passed through a softmax function to map it to the range of [0, 1], which enables channel-wise weighting of the input feature map through element-wise multiplication. Channels with higher weights are considered more significant.

In the CCAM design, we adopt softmax rather than sigmoid for channel weighting. While sigmoid is commonly used, it treats channels independently and may assign similar weights across them, limiting discrimination. Softmax instead normalizes the weights to sum to one, emphasizing the relative differences among channels and thereby supporting more effective suppression of noise and highlighting informative features. This choice is also consistent with established deep learning practices, where softmax is widely employed for normalized weighting in multi-channel scenarios [35]. To summarize, the refined feature map $F_{CA}$ by CA block is computed as

(5) $$F_{CA}\left(z,W\right)=\mathrm{Softmax}\left(W_{1}\left(W_{0}AvgPool\left(z\right)\right)\right)*z,$$

where z denotes the input feature, and $W_{0}\in R^{\frac{C}{g\times r}\times \frac{C}{g}}$ and $W_{1}\in R^{\frac{C}{g}\times \frac{C}{g\times r}}$ denote the parameters of two FC stages, in which *r*, as shown in Figure 8, stands for the reduction ratio with 16 in WeldLight, and *C* denotes the input tensor number of CCAM, while *g* stands for the number of groups (splits) for the input tensor.

In addition, CCAM adopts a cascaded group structure to further reduce the computational overhead. As illustrated in Figure 6 and Figure 8, the deepest output feature map of the backbone is split into g parts, which are then fed into the CCAM. In the CCAM, the output from each CA block is merged with the next split by element-wise addition to enhance the information fusion between channels. The process is repeated across all g groups.

For CCAM, the computation generated by the FC layer is $\frac{2C^{2}}{g\times r}$. Instead of relying on the cascaded group structure, the computation generated by the FC layer through direct utilization of channel attention leads to a calculation amount of $\frac{2C^{2}}{r}$. The computation of the CA module, when employing a cascaded group structure, is approximately $\frac{1}{g}$ of the unused capacity. However, this structure also enables multi-layer attention effects, allowing the model to refine its feature selection at each stage.

### 3.3. Loss Function

The head layer produces three outputs, a heatmap tensor $\hat{M}\in {\left[0,1\right]}^{\frac{W}{R}\times \frac{H}{R}\times 1}$, an offset tensor $\hat{O}\in {\left[0,1\right]}^{\frac{W}{R}\times \frac{H}{R}\times 2}$, and a classification tensor (i.e., one-hot encoding) $\hat{K}\in {\left[0,1\right]}^{1\times 1\times c}$, respectively, where *W* and *H* are the input image dimensions of the model, and *c* is the number of categories. Throughout the training process, the loss is determined by comparing the predictions generated by the head layer with the ground truth label values, emphasizing that the shapes of the label values should match those of the predictions. This contrast aims to optimize the neural network parameters to achieve an optimal score or regression value prediction for the model’s output. Consequently, it is essential to address the generation of labels in this context. The coordinate of the feature points location, following their mapping to the corresponding bin in the label of heatmap tensor, is $\left({\tilde{G}}_{x},{\tilde{G}}_{y}\right)=\left(\left\lfloor \frac{x_{k}}{R}\right\rfloor ,\left\lfloor \frac{y_{k}}{R}\right\rfloor \right)$, where $\left(x_{k},y_{k}\right)$ is the coordinate of the *k*-th feature point with respect to the input image size of the model (i.e., 512 × 512 for WeldLight), and R=4 denotes the downsampling stride factor. On the label heatmap, positive sample points are represented using Gaussian circles $M\left(x,y\right)=\exp\left(-\frac{{\left(x-{\tilde{G}}_{x}\right)}^{2}+{\left(y-{\tilde{G}}_{y}\right)}^{2}}{2\sigma_{p}^{2}}\right)$ to accelerate the convergence of the model [36,37], where $\sigma_{p}$ is a factor that varies with the size of the object [37], and $M\left({\tilde{G}}_{x},{\tilde{G}}_{y}\right)=1$ stands for the center of Gaussian circles, which is the ideal positive location of feature points mapping on the heatmap.

In light of the inherent rounding effect of floor division, relying solely on the heatmap to restore position information for feature points can result in accuracy loss. Offsets are introduced to address this issue. The label value of offsets of every feature point is denoted by

(6) $$O_{k}=\left(\frac{x_{k}}{R}-{\tilde{G}}_{x},\frac{y_{k}}{R}-{\tilde{G}}_{y}\right),$$

where $O_{k}$ is a 2-D coordinate representing the relative position proportion of a feature point in a corresponding bin of the label heatmap. Therefore, the offset is represented by a two-channel tensor. Additionally, the offset tensor bins are activated only at the corresponding positive locations within the heatmap tensor.

Let ${\hat{M}}_{ij}$ be the predicted score at location $\left(i,j\right)$ of the heatmap produced by WeldLight, and let $M_{ij}$ be the value at the same location on the label heatmap with Gaussian circles; a variant of focal loss [38] is used to optimize the heatmap prediction, denoted by

(7) $$L_{\mathrm{hm}}=\frac{-1}{N}\sum_{i=1}^{H}\sum_{j=1}^{W}\left\{\begin{array}{cc} {\left(1-{\hat{M}}_{ij}\right)}^{\alpha }\log\left({\hat{M}}_{ij}\right) & \mathrm{if}\;M_{ij}=1\\ {\left(1-M_{ij}\right)}^{\beta }{\left({\hat{M}}_{ij}\right)}^{\alpha }\log\left(1-{\hat{M}}_{ij}\right) & \mathrm{otherwise} \end{array}\right.,$$

where *N* is the number of feature points in a structured light stripe image, *H* and *W* stand for the spatial size of the heatmap, α is utilized to fine-tune the weights for challenging and easily locatable points, while β governs the weighting of non-central values within the Gaussian circle. Importantly, the loss weight assigned to a predicted score in $\hat{M}$ increases, as the distance from the Gaussian center in the label grows.

The essence of offset prediction is rooted in a regression task. In order to realize the accurate offset prediction, a smooth L1 loss function [39] is utilized as follows:

(8) $$L_{off}=\frac{1}{N}\sum_{i=1}^{N}\mathrm{SmoothL}1\mathrm{Loss}\left({\hat{O}}_{k},O_{k}\right),$$

where *N* is the number of feature points in a structured light stripe image, too. The smooth L1 loss leverages the advantages of both the L1 and L2 loss functions. This method prevents gradient explosion in the initial stages of training and promotes the acquisition of a more tempered gradient during back-propagation at the end of training. This contributes significantly to improved model convergence.

Cross-entropy loss (CE loss) can be applied to the weld classification task. Here, let ${\hat{K}}_{i}\in \left[0,1\right]$ represent the predicted score of structured light stripe image for class *i*, predicted by WeldLight, and boolean variable $K_{i}$ denotes the label value of class *i* in the one-hot encoding *K*; hence, the loss produced by classification is denoted by

(9) $$L_{cls}=\mathrm{CEloss}=-\sum_{i=1}^{c}K_{i}\log\left({\hat{K}}_{i}\right),$$

where *c* stands for the total number of welding seam types. Finally, the total loss $L_{total}$ is the overall training objective, which is derived as follows:

(10) $$L_{total}=L_{hm}+\lambda_{off}L_{off}+\lambda_{cls}L_{cls},$$

where $\lambda_{off}$ and $\lambda_{cls}$ represent the constant weights assigned to the offset and classification loss, configuring to a value of 1.

### 3.4. Post-Processing

**Figure 9:**
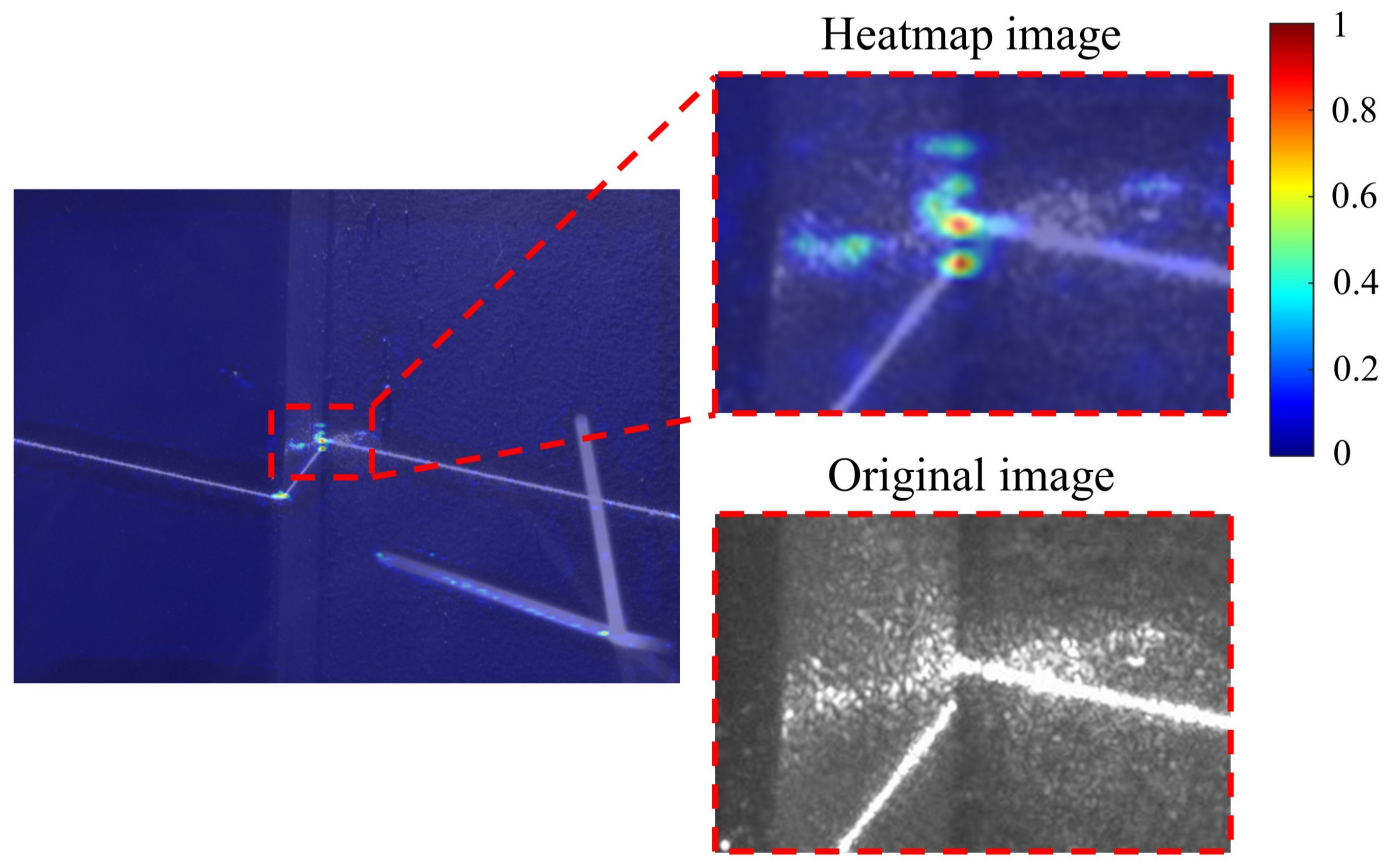
A case in which some candidate feature points may not be filtered out when the pool filter is small.

A correlation exists between the prediction of point numbers and laser stripe classification, as discussed in [11]. Specifically, if the classification result is predicted as “lap-seam,” the model identifies the first two bins on the heatmap tensor with the highest predicted scores. These are then combined with the corresponding bins on the offset tensor, leading to the identification of the final predicted positions. In this scenario, there is no need to consider the suppression by predicted score thresholds. However, as depicted in Figure 9, the heatmap tensor undergoes resizing to match the uniform shape of the original image, covering it entirely. The colors in the heatmap indicate the predicted scores assigned to the potential feature points.

Notably, the figure illustrates a broken laser stripe, where both ends are confidently identified as potential feature points by WeldLight, despite their proximity. An empirical solution to this issue involves applying a 5×5 max-pooling operation to the heatmap. Bins with unchanged values correspond to effective potential feature points by comparing the heatmap tensor before and after pooling. In cases of closely located potential feature points, only the one with the highest predicted score is retained, effectively resolving the issue.

## 4. Verification

### 4.1. Test Environment

The network was constructed using the PyTorch 2.0.0 deep learning framework based on Python 3.8. The dataset utilized for training and testing the model was obtained from the welding seam positioning and tracking sensor mentioned earlier. The model was trained on a computer with an Nvidia 2080Ti GPU and an Intel Xeon (R) E5-2683 CPU. The model was converted to the ONNX format and used OpenVINO for DNN inference on an EIC with an Intel Core i7-8650U CPU. The network initializes its backbone with the weights of MobileNetv3 pretrained on ImageNet to expedite training and achieve rapid convergence. The FC layer was implemented using a standard 1 × 1 convolution, and the convolution layer parameters were initialized using the He initialization method. As for the BN layer parameters, the weights were initialized to one, and the biases were initialized to zero.

The initial learning rate was set to $5\times 10^{-4}$ using Adam as the optimizer (momentum = 0.9, weight decay = 0), and a cosine annealing strategy was adopted for learning rate scheduling. The batch size of the training process was set to 32. The maximum number of training epochs was set to 500, and an early stopping strategy was applied such that the training was terminated at the 50th epoch once the validation loss had stabilized. These hyperparameter values were determined empirically to balance convergence speed and training stability.

Upon observing convergence in the loss function of the validation set during the training stage, the model was utilized to evaluate both the positioning performance of the feature points and the classification performance of the welding seams. The comparative model opted for YOLOv5n and DETR, trained on the COCO dataset, to obtain initial weights, which were subsequently fine-tuned using the default training configurations provided by the respective open-source projects.

### 4.2. Weld Classification Performance

For welding tasks, the baseline network used in WeldLight is CenterNet, which, similar to DETR and YOLOv5n employed for comparison, belongs to the family of object detection networks. Since a welding image should contain only a single class, previous studies have commonly adopted the class of the point with the highest classification confidence as the image-level label. WeldLight, however, incorporates a dedicated prediction branch to directly infer the class of the entire image rather than distinguishing the classes of individual feature points, thereby better adapting to the requirements of weld seam classification in welding operations. In contrast, DETR, through its end-to-end prediction mechanism, is able to model the relationship between the global image context and the classification of feature points, thereby reducing the risk of assigning multiple classes to different points within the same image. By design, YOLOv5n assigns a class label to each detected point, which necessitates determining the overall image category based on the point with the highest classification confidence.

**Figure 10:**
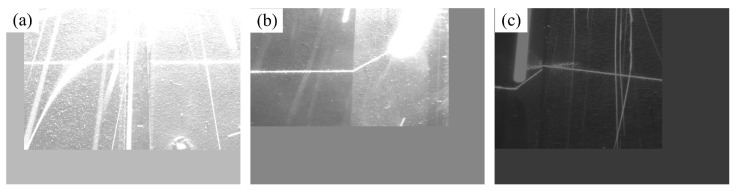
Part of the pictures in the adjusted test set used in the performance test of welding classification. (**a**–**c**) are examples of the adjusted Y-seam, lap-seam, and butt-seam images in the classification performance test.

Notably, all three models accurately classified 150 images in the test set. In pursuit of a more granular comparison, the images underwent adjustments, including random cropping, translation, deformation, and brightness modification. For a visual representation, a selection of the test set images utilized in the classification performance evaluation is presented in Figure 10.

**Figure 11:**
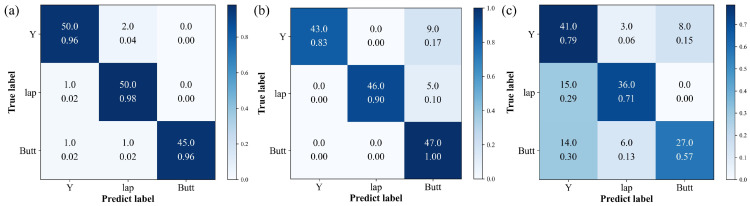
Confusion matrix obtained by different models on the adjusted test set. (**a**–**c**) correspond to the prediction results of WeldLight, YOLOv5n, and DETR, respectively.

The adjusted test set was used to evaluate the three models, respectively. Finally, the classification performance was evaluated by the confusion matrix obtained by each model, presented in Figure 11.

The core of the weld classification involves a multi-classification task. To streamline comparisons and address discrepancies in sample numbers, the evaluation of the three models in welding seam classification employed the following three metrics:

(11) $$P_{w}=\sum_{i=1}^{N}w_{i}P_{i}=\sum_{i=1}^{N}w_{i}\frac{TP_{i}}{TP_{i}+FP_{i}}$$

(12) $$R_{w}=\sum_{i=1}^{N}w_{i}R_{i}=\sum_{i=1}^{N}w_{i}\frac{TP_{i}}{TP_{i}+FN_{i}}$$

(13) $$F1_{w}=\sum_{i=1}^{N}w_{i}\frac{2P_{i}\cdot R_{i}}{P_{i}+R_{i}}$$

where $P_{w}$ $R_{w}$ $F1_{w}$ stand for the weighted-average precision, recall, and F1-score, respectively. $P_{i}$, $R_{i}$, and $F1_{i}$ are the precision, recall, and F1-score of class *i*, respectively. $w_{i}$ represents the ratio of the sample size of category i to the total sample size. True positive (TP), false negative (FN), and false positive (FP) are values that can be determined from the confusion matrix [40]. Table 2 displays the metric values of the three models on the adjusted test set. Notably, WeldLight exhibited the highest values across the three key metrics assessing classification performance, with a weighted-average precision of 0.9674, a weighted-average recall of 0.9666, and a weighted-average F1-score of 0.9668.

### 4.3. Feature Point Positioning Performance

**Figure 12:**
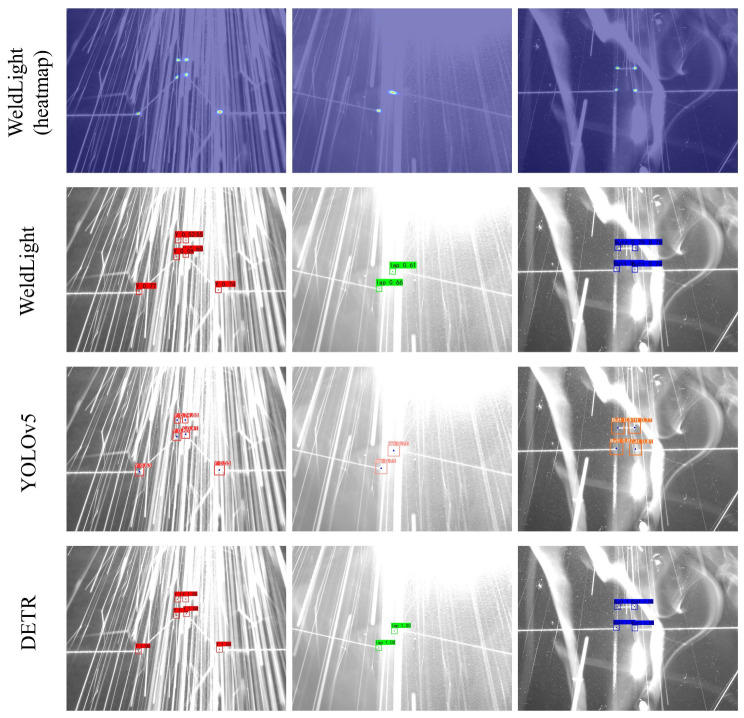
Part of the regression results obtained by different models on the test set.

Welding-related noise, such as splash, reflection, and smog, can compromise the quality of images captured by industrial cameras. Figure 12 demonstrates that all three models reliably distinguish laser stripes from noise and precisely localize feature points, maintaining robust performance even under noise conditions.

To evaluate the robustness under different noise levels, the test dataset was divided into high-noise and low-noise subsets, each accounting for 50% of the samples. After post-processing, the three models were evaluated for their feature point positioning performance on both subsets. The predictions were generated for the coordinates of the feature points with respect to the original image resolution of 1440×1080. The purpose of this evaluation is to assess the models’ feature point positioning accuracy under varying noise conditions and to analyze their noise robustness. The performance metrics include the mean absolute error (MAE), root mean square error (RMSE), standard deviation ($\sigma_{f}$), and projecting the predicted coordinates and the label coordinates to compute the mean Euclidean distance ($\rho_{mean}$) with respect to the CCF. The positioning errors of the feature points were calculated along both the *X*-axis and *Y*-axis of the image, as follows:

(14) $$e_{i}=\left\{\begin{array}{cc} q_{xi}-{\hat{q}}_{xi} & \mathrm{if}\;\mathrm{axis}\;=\;x\\ q_{yi}-{\hat{q}}_{yi} & \mathrm{if}\;\mathrm{axis}\;=\;y \end{array}\right.,$$

where $\left(q_{xi},q_{yi}\right)$ and $\left({\hat{q}}_{xi},{\hat{q}}_{yi}\right)$ stand for the coordinate of the label and the predicted coordinate of the feature points, respectively.

The MAE was used to evaluate the static precision of the positioning, which is derived as follows:

(15) $$\mathrm{MAE}=\frac{1}{N}\sum_{i=1}^{N}∥e_{i}∥,$$

where *N* denotes the total number of feature points in the designated test set and keeps the same definition in the following formulas.

The RMSE would highlight the impact of predicted outliers, which is derived as follows:

(16) $$\mathrm{RMSE}=\sqrt{\frac{1}{N}\sum_{i=1}^{N}e_{i}^{2}}.$$

$\sigma_{f}$ was used to evaluate the stability/robustness of the model [7], which is derived as follows:

(17) $$\sigma_{f}=\sqrt{\frac{1}{N-1}\sum_{i=1}^{N}{\left(e_{i}-{\bar{e}}_{i}\right)}^{2}},$$

where ${\bar{e}}_{i}$ is the average value of $e_{i}$ at all feature points.

The metric $\rho_{mean}$ was employed to assess the error level of three models after projecting 2-D positioning results into the 3-D space. It is derived as follows:

(18) $$\rho_{mean}=\frac{1}{N}\sum_{i=1}^{N}\sqrt{{\left(p_{xi}-{\hat{p}}_{xi}\right)}^{2}+{\left(p_{yi}-{\hat{p}}_{yi}\right)}^{2}+{\left(p_{zi}-{\hat{p}}_{zi}\right)}^{2}},$$

where ($p_{xi},p_{yi},p_{zi}$) and (${\hat{p}}_{xi},{\hat{p}}_{yi},{\hat{p}}_{zi}$), which stand for the label and predicted coordinates of feature points with respect to CCF, can be calculated by Equation (3), based on $\left(q_{xi},q_{yi}\right)$ and $\left({\hat{q}}_{xi},{\hat{q}}_{yi}\right)$. An a priori assumption is made that the calibration result of the line structured light plane equation and the intrinsic matrix of the camera is ideal, attributing any error solely to inaccuracies in the feature points’ locations within the PCF, as predicted by the positioning models.

**Figure 13:**
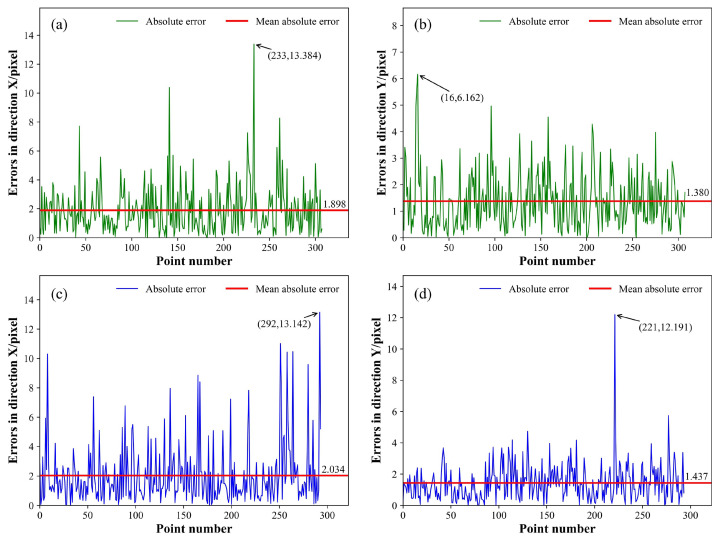
The absolute error curve of the WeldLight. (**a**,**b**) illustrate the feature points regression absolute errors of the model in the X and Y directions, respectively, evaluated on the low-noise test set. (**c**,**d**) showcase the feature points regression absolute errors of the WeldLight in the X and Y directions, respectively, tested on the high-noise test set. The peak value, the peak position of the absolute error, and the MAE are annotated.

**Figure 14:**
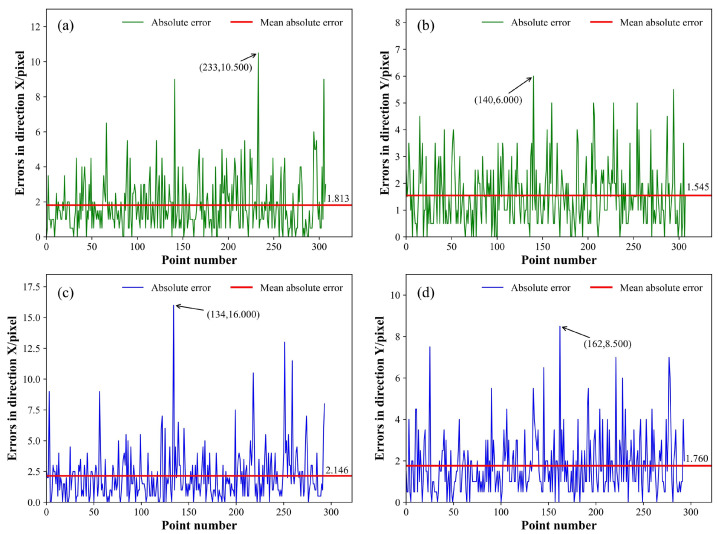
The absolute error curve of the YOLOv5. (**a**,**b**) illustrate the feature points regression absolute errors of the model in the X and Y directions, respectively, evaluated on the low-noise test set. (**c**,**d**) showcase the feature points regression absolute errors of the YOLOv5 in the X and Y directions, respectively, tested on the high-noise test set. The peak value, the peak position of the absolute error, and the MAE, are annotated.

**Figure 15:**
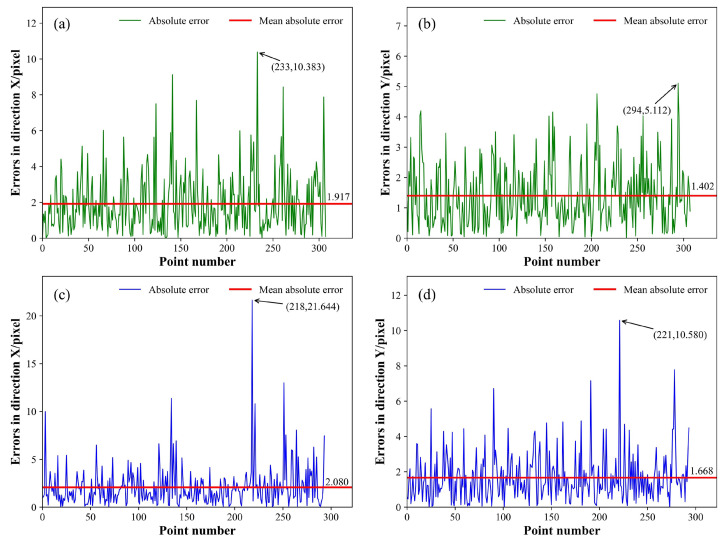
The absolute error curve of the DETR. (**a**,**b**) illustrate the feature points regression absolute errors of the model in the X and Y directions, respectively, evaluated on the low-noise test set. (**c**,**d**) showcase the feature points regression absolute errors of the DETR in the X and Y directions, respectively, tested on the high-noise test set. The peak value, the peak position of the absolute error, and the MAE are annotated.

The absolute error curve, which represents the test outcomes using three models, is depicted in Figure 13, Figure 14 and Figure 15, respectively. The green curve corresponds to the absolute error measured from the low-noise test set, the blue curve corresponds to absolute error measurements from the high-noise test set, and the red horizontal line denotes the MAE. All metrics related to the positioning errors of the feature points were calculated and arranged in Table 3 and Table 4; the MAE, RMSE, and $\sigma_{f}$ were assessed along the X and Y directions of the image, and the average values along the two directions were calculated, represented as the average-MAE, average-RMSE, and average-$\sigma_{f}$. Simultaneously, the outcomes of WeldLight without CCAM are included in Table 3 and Table 4, too. Notably, there was no specialized positioning performance analysis for different weld types in the test set due to the inherent uncertainty in weld types during welding processes.

The outcomes of the positioning performance evaluation for the weld feature points on a low-noise test dataset are listed in Table 3. Within the low-noise scenario, the three models are basically at the same level for the MAE and RMSE. Notably, regarding the average-$\sigma_{f}$, WeldLight with and without CCAM exhibited outstanding performance compared to the other two models, achieving values of 1.943 pixels and 1.922 pixels, respectively. In terms of $\rho_{mean}$, WeldLight showed the best performance, reaching 0.197 mm. The utilization of CCAM did not yield a significant difference in the low-noise test set condition.

The experimental findings from the positioning performance assessment of the welding seam feature points in a high-noise test set are illustrated in Table 4. Within the high-noise environment, WeldLight exhibited an average MAE, RMSE, and $\rho_{mean}$ of 1.736 pixels, 2.407 pixels, and 0.205 mm, respectively. These metrics outperformed both YOLOv5 and DETR. Moreover, compared to WeldLight without CCAM, the model integrating CCAM showcased enhanced performance across nearly all the positioning metrics, reflecting more stable and accurate localization results. This improvement suggests that CCAM contributes to filtering out noise interference and enhancing the robustness of feature extraction, which is particularly beneficial in high-noise welding scenarios. This phenomenon contrasts the measurement results observed in the low-noise test set. WeldLight excels in average $\sigma_{f}$, registering a value of 2.217 pixel, underscoring WeldLight’s superior stability in accurately localizing weld feature points within high-noise welding images.

### 4.4. Lightweight Level

The model’s lightweight characteristics were evaluated based on multiple metrics, including the total number of model parameters (Params), floating point operations (FLOPs), mean latency, and frames per second (FPS). Params serve as an indirect measure of the computational complexity and memory utilization, while FLOPs represent the computational cost of the model [41]. A lower mean latency (or a higher FPS) implies reduced inference time, a critical factor for optimal real-time performance in the welding seam tracking system employing this model. This aspect is particularly crucial for complex seam tracking applications, welding speed, and welding process control. The mean latency of the three models was determined through 100 tests using a single image, and the frames per second (FPS) value was calculated by dividing 1000 ms by the mean latency.

It is worth noting that, despite a consistent evaluation criterion, DETR was not designed for real-time tasks and thus lacks advantages in inference speed. Moreover, Transformer-based architectures such as DETR face inherent challenges in learning low-level features from scratch on limited datasets, making large-scale pretraining essential to achieve competitive performance with CNNs [42]. In this study, these limitations were mitigated by pretraining on the COCO dataset, followed by local fine-tuning until full convergence. Nevertheless, such architectural characteristics remain important considerations when interpreting the results. Naturally, these factors were also taken into account during the initial design of WeldLight, to avoid similar limitations.

Table 5 provides an overview of the lightweight metrics for the three models. WeldLight outperformed the others across all metrics, with the Params being 1.3 M, translating to 72% of YOLOv5n, 3.5% of DETR, and 0.87 GFLOPs, representing 21% of YOLOv5n and 1.5% of DETR. With a mean latency of 29.32 ms and FPS at 34.11 Hz, WeldLight fulfills stringent requirements for real-time weld seam tracking.

## 5. Conclusions

1. An improved CenterNet framework was proposed for positioning welding light-stripe feature points. The average MAE in the X and Y directions was 1.639 and 1.736 pixels, respectively, on both low- and high-noise test sets, demonstrating high prediction accuracy. On a single-line structured light platform, the mean Euclidean distance ($\rho_{mean}$) of the 3-D feature point coordinates in the CCF was 0.197 mm and 0.205 mm for the two test sets, further confirming the robustness of the proposed method.
2. In the context of welding seam classification, an approach was proposed that directly derived one-hot encoding for welding seam classification predictions from coarse-resolution features selected by the attention module. This method differed from the conventional method of upsampling to the high-resolution features for prediction. WeldLight achieved an F1-score of 0.9668 on the adjusted test set.
3. An online data augmentation method, focused on randomly generated splash noise, was proposed to adapt the model to natural splash noise arising from welding processes, thereby reducing its dependence on the quality of the dataset. Furthermore, a lightweight attention module, CCAM, was introduced as a feature selector to enhance the model’s robustness against welding noise. The results show that WeldLight exhibited improved performance, particularly in high-noise scenes, upon incorporating CCAM.
4. The model was deployed on an EIC, utilizing the CPU for inference. The average processing speed for a single image was 29.32 ms, translating to 34.11 Hz, thereby satisfying the real-time requirements of the seam tracking system.

This study, rooted in real-world applications, reduces the computational and dataset requirements, thereby providing manufacturers with practical guidelines for deploying weld-seam tracking on edge devices without the need for costly GPUs. This capability not only lowers the deployment costs but also enhances automation and reduces the reliance on skilled labor.

Nevertheless, some limitations remain. Future research will focus on evaluating the model in more diverse welding scenarios and employing statistical methods to strengthen the reliability of the results. Moreover, the current architecture still has room for refinement; integrating advanced lightweight designs and exploring Transformer- or Mamba-based frameworks may further improve the performance. Finally, because the present approach still relies on manual annotation, future studies will investigate unsupervised and semi-supervised strategies to better adapt to evolving welding environments.

## Figures and Tables

**Table 1:** Partition of the dataset.

Dataset	Y	Lap	Butt	Total
Train	415	404	396	1215
Val	33	45	57	135
Test	52	51	47	150
Total	500	500	500	1500

**Table 2:** Test overall metric scores of different models on adjusted test set.

	WeldLight	YOLOv5n	DETR
$P_{w}$	**0.9674**	0.9281	0.9336
$R_{w}$	**0.9666**	0.9067	0.9333
${F1}_{w}$	**0.9668**	0.9090	0.9334

**Table 3:** Statistical characteristics of the positioning performance of the three models for light stripe feature points of the low-noise weld structure. ^†^: WeldLight omited the utilization of CCAM. The units for MAE, RMSE, and $\sigma_{f}$ were all expressed in pixels, while the $\rho_{mean}$ was measured in mm.

	Ours	Ours ^†^	YOLOv5n	DETR
X-MAE	1.898	1.859	**1.813**	1.917
Y-MAE	**1.380**	1.502	1.545	1.402
Average-MAE	**1.639**	1.681	1.679	1.660
X-RMSE	2.544	**2.326**	2.401	2.535
Y-RMSE	**1.740**	1.885	1.926	**1.740**
Average-RMSE	2.142	**2.106**	2.164	2.138
X-$\sigma_{f}$	2.258	**2.183**	2.392	2.422
Y-$\sigma_{f}$	**1.627**	1.660	1.911	1.730
Average-$\sigma_{f}$	1.943	**1.922**	2.152	2.076
$\rho_{mean}$	**0.197**	0.216	0.232	0.209

**Note:** The best results are highlighted in bold.

**Table 4:** Statistical characteristics of the positioning performance of the three models for light stripe feature points of a high-noise weld structure. ^†^: WeldLight omited the utilization of CCAM.

	Ours	Ours ^†^	YOLOv5n	DETR
X-MAE	**2.034**	2.123	2.146	2.080
Y-MAE	**1.437**	1.646	1.760	1.668
Average-MAE	**1.736**	1.885	1.953	1.874
X-RMSE	2.931	**2.927**	3.023	3.043
Y-RMSE	**1.883**	2.206	2.275	2.172
Average-RMSE	**2.407**	2.567	2.649	2.608
X-$\sigma_{f}$	**2.642**	2.731	3.029	2.919
Y-$\sigma_{f}$	**1.792**	2.032	2.254	2.176
Average-$\sigma_{f}$	**2.217**	2.382	2.646	2.548
$\rho_{mean}$	**0.205**	0.231	0.253	0.238

**Note:** The best results are highlighted in bold.

**Table 5:** Comparison of lightweight metrics of three models on Intel Core i7-8650U CPU.

	WeldLight	YOLOv5n	DETR
Params (M)	**1.3**	1.8	37
GFLOPs	**0.87**	4.1	57
Mean latency (ms)	**29.32**	53.07	681.43
FPS (Hz)	**34.11**	18.83	1.47

**Note:** The best results are highlighted in bold.

## Data Availability

The raw data supporting the conclusions of this article will be made available by the authors on request.
